# Antibody-mediated SARS-CoV-2 entry and conformational regulation

**DOI:** 10.1128/spectrum.00120-26

**Published:** 2026-08-21

**Authors:** Casey L. Kiyohara, Sheamin Kim, Wendy E. Thomas

**Affiliations:** 1 Department of Bioengineering, University of Washington 7284, Seattle, Washington, USA; 2 The Information School, University of Washington 7284, Seattle, Washington, USA; Instituto Nacional de Tecnologia Agropecuaria, Buenos Aires, Argentina

**Keywords:** SARS-CoV-2, antibody-dependent enhancement, antibody

## Abstract

Antibody-mediated entry (AME) of severe acute respiratory syndrome coronavirus 2 (SARS-CoV-2) into monocytes and macrophages has been linked to activation of inflammatory phenotypes that are associated with severe coronavirus disease 2019 (COVID-19), but why only some antibodies mediate this entry while others do not is unknown. However, it has been demonstrated that conformational dynamics of the SARS-CoV-2 receptor-binding domain (RBD) are critical to viral entry, and that antibodies targeting the RBD can conformationally regulate these dynamics. Here, we identified four groups of RBD-specific monoclonal antibodies (mAbs) that target unique epitopes on the SARS-CoV-2 RBD and are also associated with different RBD conformational regulation and AME abilities. Steric clash quantification elucidated a structural basis for differences in conformational regulation by these antibody groups. We found that some, but not all, antibody groups were able to mediate entry into THP1-derived macrophages, and also found that this entry was inhibitable by antibodies in other groups. Collectively, these results suggest that there is a connection between RBD conformational regulation, epitope, and AME for antibodies targeting SARS-CoV-2. This connection improves understanding of the AME mechanism and how to inhibit AME for SARS-CoV-2, which has potential applications in the continued development of safe and effective vaccines and therapeutics for COVID-19 and related diseases.

**IMPORTANCE:**

Antibody-mediated entry (AME) of severe acute respiratory syndrome coronavirus 2 (SARS-CoV-2) into immune cells and resulting activation of inflammatory pathways is increasingly being associated with coronavirus disease 2019 (COVID-19) disease severity. Vaccination against COVID-19 remains one of our strongest tools to prevent these effects; vaccines continue to protect patients from both severe and long COVID-19 symptoms, and serum from vaccinated individuals was shown not to cause AME in immune cells *in vitro*. However, a lack of understanding about why some, but not all, antibodies cause AME limits our ability to actively prevent AME in antigen design. In this work, we identify characteristics that differentiate individual AME and non-AME antibodies. We also report that AME can be inhibited by other antibodies. Understanding these characteristics and differences between antibodies could allow us to add functionality to already effective vaccines by designing them to actively avoid eliciting AME antibodies and promote antibodies that inhibit AME and its downstream systemic effects.

## INTRODUCTION

Despite the rapid development and administration of vaccines to protect against coronavirus disease 2019 (COVID-19), the pandemic continues to persist globally. Continued spread of severe acute respiratory syndrome coronavirus 2 (SARS-CoV-2), the virus that causes COVID-19, is driven in part by the emergence of highly transmissible and immune-evasive variants of the virus (1). Severe COVID-19 often presents with a macrophage activation syndrome (MAS)-type phenotype, which is characterized by the release of high levels of inflammatory cytokines, including IL-1, IL-6, TNF-α, G-CSF, and CXCL10, by immune cells (2, 3). In COVID-19, this hyperinflammatory cytokine storm ultimately leads to effects such as pulmonary intravascular coagulopathy and acute respiratory distress syndrome (ARDS) (2, 3).

There is evidence that SARS-CoV-2 infection of FcγR-expressing cells, particularly angiotensin-converting enzyme 2-(ACE2-)negative monocytes and macrophages, occurs (4–6) and is relevant to inflammation associated with COVID-19 (7). Infected ACE2-negative monocytes and lung macrophages isolated from COVID-19 patients have shown activation of inflammasomes, which cause release of IL-1 cytokines and initiation of pyroptosis, an inflammatory form of cell death that leads to further cytokine release and immune cell recruitment (7). Notably, key pyroptosis markers were particularly elevated for severe COVID-19 patients relative to patients with mild or moderate disease (7). Several viruses, such as Dengue, HIV, and Ebola, have demonstrated antibody-dependent enhancement (ADE) of viral infection, where subneutralizing levels of antibodies targeting the virus facilitate entry into and production of live virus inside FcγR-expressing cells by allowing binding to FcγRs (8). It has been shown that, in contrast, while SARS-CoV-2 can undergo antibody-mediated entry (AME) into FcγR-expressing cells (7, 9–12), this infection is abortive, not leading to replication of live virus inside the cell (7, 13). Nevertheless, given the key role of cytokine storms in COVID-19 disease (2, 3), prior work demonstrating SARS-CoV-2 infection and subsequent inflammation and cytokine release for ACE2-negative macrophages and monocytes from COVID-19 patients supports the clinical relevance of AME even without productive internal viral replication.

Understanding the mechanism of AME into FcγR-expressing cells is critical to preventing it and its downstream inflammatory effects (7). It has been shown that AME via FcγRIIb is blocked by inhibitors of macropinocytosis, endocytosis, phosphoinositide 5-kinase activity, and lysosome acidification (10). AME therefore likely requires not only viral binding to FcγRs, but also endocytosis of the antibody-viral complex, followed by spike cleavage and viral fusion (10). Although AME of SARS-CoV-2 into FcγR-expressing cells has been demonstrated for both monoclonal antibodies (mAbs) (9, 10, 12) and COVID-19 patient serum (14, 15), why some antibodies and serum mediate entry, and others do not, is not fully understood. SARS-CoV-2 AME antibodies have, to our knowledge, all been shown to neutralize entry into ACE2-expressing cells (9, 10, 12) and most, but not all (12), bind the SARS-CoV-2 RBD (9, 10). Afucosylation (7) and epitope (12) have been implicated as potential differentiating antibody characteristics, and antibodies seem to require bivalent interactions with the spike to mediate entry via FcγRIIb (10). Notably, it is unknown whether or how the conformational changes of the SARS-CoV-2 spike that are associated with viral fusion with ACE2-expressing cells (16) are involved in antibody-mediated viral fusion with FcγR-expressing cells.

SARS-CoV-2 initiates cell infection by binding to ACE2 on host cells via its spike protein (16, 17). The SARS-CoV-2 spike protein is a homotrimer, with each protomer consisting of a receptor-binding domain (RBD)-containing S1 subunit and a membrane-anchored and fusion-mediating S2 subunit (16–18). In the unbound prefusion conformation, the SARS-CoV-2 spike protein RBDs change between two conformational states: up, or open, where the ACE2 binding site is exposed, and down, or closed, where the ACE2 binding site is buried (18). For unbound SARS-CoV-2 spike trimers, there is an equilibrium between a state with all three RBDs in the down conformation (3-down) and a state with two RBDs in the down conformation and one in the up conformation (1-up) (17, 18). Binding of the S1 subunit to ACE2 exposes a cleavage site that, upon cleavage, causes the spike S1 subunit to be released, or shed, from the S2 subunit (18). The S2 subunit then undergoes significant conformational changes into a postfusion conformation, including an intermediate conformation that initiates fusion of the viral and cell membranes (18). Notably, it has been shown that monoclonal antibodies can affect the conformational dynamics of the SARS-CoV-2 RBD and spike in multiple ways that are likely to affect viral entry into ACE2-expressing cells (9, 10, 19–24). We hypothesize that differences in this RBD conformational regulation determine which antibodies mediate SARS-CoV-2 entry and fusion with FcγR-expressing cells.

Here, we investigate antibodies that regulate the conformational dynamics of the SARS-CoV-2 RBD in a variety of ways and the relationship between this conformational regulation, AME, and the antibody epitope, which has also been shown to correlate with AME (12). Understanding these factors that may affect AME will help improve understanding of the AME mechanism and could drive future development of AME inhibitors and non-AME-inducing therapeutics and vaccines. This approach could improve treatment and care for COVID-19 and diseases caused by related viruses and mechanisms.

## RESULTS

### Epitope analysis of anti-SARS-CoV-2 antibody groups

From the literature, we identified neutralizing anti-SARS-CoV-2 mAbs with unique heavy and light chain subclasses that were previously analyzed by cryo-electron microscopy (cryo-EM) or crystallography and/or analyzed for AME (9, 10, 19, 21, 24, 25). To investigate the relationship between conformational regulation and AME, we categorized the mAbs into four groups based on their conformational regulation of the SARS-CoV-2 RBD in the structural data and their ability to mediate AME (Table 1). Antibodies with “up-exclusive” behavior were identified as those that were reported to bind only to up RBDs in cryo-electron microscopy (cryo-EM) or that showed binding to an epitope buried in the down RBD conformation in x-ray crystallography structures. Antibodies with “down-exclusive” behavior were defined as those that were found to bind only down RBDs in cryo-EM studies, despite their potential epitope accessibility in the up conformation. Group 1 and Group 2 antibodies were defined as up-exclusive and down-exclusive antibodies, respectively, that did not mediate entry into FcγR-expressing cells (10) or had not been previously tested for independent AME ability. Groups 3 and 4 were differentiated largely by their respective abilities to mediate entry into cells expressing FcγRIIb (Group 4) or both FcγRIIb and FcγRI (Group 3). Their conformational regulation abilities were less defined by previous work than those of Groups 1 and 2, but one Group 3 antibody showed up-exclusive behavior and was reported to interact with an adjacent up RBD (9). Group 4 antibodies were shown to shift the spike trimer conformational equilibrium away from the 3-down state toward a range of 1-, 2-, and 3-up states, and were therefore classified as “up-preferring” (9, 10). From this analysis, we thus identified four unique antibody groups that share conformational regulation and AME characteristics. Notably, no AME antibodies that had been previously characterized structurally showed down-exclusive conformational regulation.

**TABLE 1:** Unique antibody epitopes are associated with different conformational and AME effects for SARS-CoV-2. The antibodies were grouped by conformational regulation and AME characteristics

mAb group*^a^*	mAb name	Conformational regulation^*b*^	AME*^c^*	PDB ID*^d^*	Heavy/light chain subclass^*e*^	Neutralizing	# Shared residues/# total residues*^f^*
1	CV30	Up-exclusive (21)	Unknown/none	*6XE1 (21)	IGHV3-53, IGKV3-20 (21)	Yes (21)	27/38
	C105	Up-exclusive (25)	Unknown/none	6XCM (25)	IGHV3-53, IGLV2-8 (25)	Yes (26)	27/34
	MW07	Up-exclusive (10)	None^*g*^ (10)	*7DK2 (10)	IGHV3-7, IGKV1-NL1 (10)	Yes (10)	27/46
2	2-4	Down-exclusive (24)	Unknown/none	6XEY (24)	IGHV1-2, IGLV2-8 (24)	Yes (24)	19/20
	C144	Down-exclusive (19)	Unknown/none	7K90 (19)	IGHV3-53, IGLV2-14 (19)	Yes (26)	19/27
3	DH1047	Up-exclusive (9)	FcγRI and FcγRIIb^*h*^ (9)	7LD1 (9)	IGHV1-46, IGKV4-1 (9)	Yes (9)	Unknown/37
	DH1046	Unknown	FcγRI and FcγRIIb^*h*^ (9)	n/a	IGHV3-23, IGKV1-5 (9)	Yes (9)	Unknown
4	DH1041	Up-preferring (9)	FcγRIIb^*h*^ (9)	7LAA (9)	IGHV3-7, IGLV1-40 (9)	Yes (9)	17/28
	DH1043	Up-preferring (9)	FcγRIIb^*h*^ (9)	7LJR (9)	IGHV1-69, IGKV3-20 (9)	Yes (9)	17/33
	MW01	Unknown	FcγRIIb^*g*^ (10)	*7DJZ (10)	IGHV1-69, IGKV3-15 (10)^^^	Yes (10)	17/31
	MW05	Unknown	FcγRIIb^*g*^ (10)	*7DK0 (10)	IGHV1-69, IGKV3-15 (10)^^^	Yes (10)	17/32

^*a*^ Antibodies grouped by conformational regulation (Groups 1 and 2) and AME (Groups 3 and 4).

^*b*^ Antibodies classified as up-exclusive if they were reported as only being able to bind the up RBD conformation, either through cryo-EM or through structure alignment. Antibodies classified as down-exclusive if they were reported to only bind the down conformation in cryo-EM studies. Antibodies classified as up-preferring if they were reported to prefer the up conformation in cryo-EM studies but were not reported to require it in order to bind. Antibodies classified as unknown if no structure was determined or if the crystal structure of the antibody was not reported to exclude or prefer binding to up or down RBDs.

^*c*^ Receptor through which AME was reported for each antibody. Antibodies classified as unknown were, to our knowledge, never tested for independent AME ability prior to this study. Antibodies classified as none were tested and showed no evidence of AME, either in prior studies or (when co-listed with Unknown), in this study.

^*d*^ PDB IDs for each antibody, if available. Structures were determined using cryo-EM unless marked with an asterisk (*), which indicates the structure was determined using x-ray crystallography.

^*e*^ Heavy and light chain subclasses as previously reported, were gathered using CoV-AbDab (27). While the two antibodies marked with the up arrow (^) have the same variable (V) genes, as shown, they have unique heavy and light joining (J) genes (27).

^*f*^ Number of residues shared by all antibodies in the corresponding group/number of residues in that antibody’s full epitope.

^*g*^ AME and FcγR-dependence confirmed in prior work using a combination of FcγR inhibitors, antibody mutations, testing on cell types with varying FcγR expression, and cell line engineering.

^*h*^ AME and FcγR-dependence confirmed in prior work using engineered cell lines expressing or lacking specific FcγRs.

We then examined the published structures of each antibody described in Table 1 to identify their structural epitopes and determine any epitope similarities for antibodies within groups defined by shared conformational regulation and AME characteristics. The groups that included more than one antibody structure (i.e., Groups 1, 2, and 4) each showed an intra-group overlap of residues shared among all antibody epitopes in the respective group. This epitope overlap ranged from 60.7% shared among all antibodies in Group 4 (17 shared residues out of 28 total residues in the smallest epitope in that group) to 95% shared for the antibodies in Group 2 (19 shared residues out of 20 total). We therefore found that antibodies grouped by conformational regulation and AME share similar epitopes.

**Fig 1:**
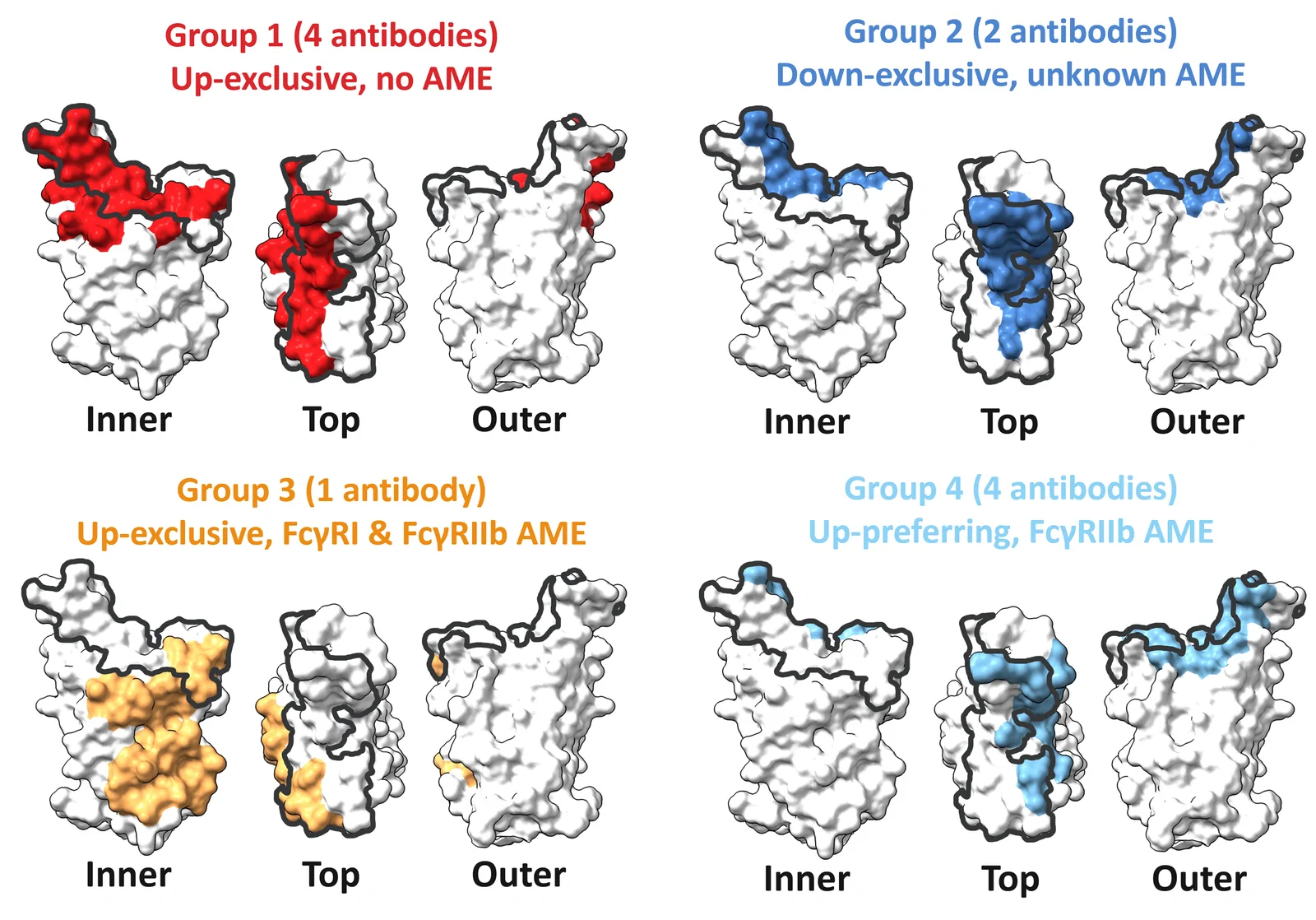
Shared epitopes are unique for antibody groups with varying conformational regulation and AME. For each group (as defined in Table 1), residues shared by all antibodies in the group are shown in color for three different views of the SARS-CoV-2 RBD. The ACE2 epitope is outlined in black. The inner face of the RBD is masked in the down RBD conformation but exposed in the up conformation; the outer face is exposed in both conformations.

The shared epitopes for each group were then mapped onto the SARS-CoV-2 RBD and compared to each other and to the ACE2 epitope (Fig. 1; Table 2). The Group 1 epitope overlapped with over half of the ACE2 binding site, with the majority of both the overlapping and non-overlapping Group 1 epitope residues lying on the inner face of the RBD that is buried when the RBD is down. Given the up-exclusive binding of both ACE2 and the Group 1 antibodies, this shared epitope and high involvement of the inner face in binding are both expected. The Group 3 FcγRI AME antibody similarly bound the inner face but overlapped much less with the ACE2 epitope than the Group 1 antibodies did, instead targeting below the ACE2 binding site. Group 3 antibody DH1046 (9) did not have an existing structure, but was previously found to compete with Group 3 AME antibody DH1047 (9), indicating that there is some overlap between their epitopes.

**TABLE 2:** Number of residues shared between antibody groups (defined in Table 1) or with ACE2^*a*^

Epitope group	1 (Up-exclusive)	2 (Down-exclusive)	3 (FcγRI & FcγRIIb AME)	4 (FcγRIIb AME)	ACE2
1 (Up-exclusive)	**27**	8	7	2	18
2 (Down-exclusive)	8	**19**	0	10	14
3 (FcγRI & FcγRIIb AME)	7	0	**37**	0	6
4 (FcγRIIb AME)	2	10	0	**17**	8
ACE2	18	14	6	8	**30**

^*a*^ Numbers are bolded when they represent the full epitope size for the indicated group, rather than the overlap between two groups.

Notably, the Group 3 and Group 4 epitopes, which represent antibodies that mediate viral entry via both FcγRIIb and FcγRI and only FcγRIIb, respectively, had no overlapping residues. The Group 4 FcγRIIb AME epitope had minimal involvement of the inner face and therefore minimal overlap with epitopes of both Group 1 and Group 3, focusing instead on the top and outer faces. The Group 2 down-exclusive antibodies had a relatively similar epitope to that of Group 4. However, the Group 2 epitope had 75% more overlap with the ACE2 binding site (14 residues) than the Group 4 epitope did (8 residues), despite the two groups having similar epitope sizes. The Group 2 epitope thereby targeted the inner and top faces more and the outer face less. Notably, the Group 2 antibodies also overlapped more with the ACE2 epitope than with the epitope of Group 4 antibodies, further suggesting that these two groups target different epitopes. Overall, this analysis suggests that antibody groups with different conformational regulation and AME characteristics target unique epitopes.

### Analysis of steric clashing for anti-SARS-CoV-2 antibodies aligned with spike trimers

**Fig 2:**
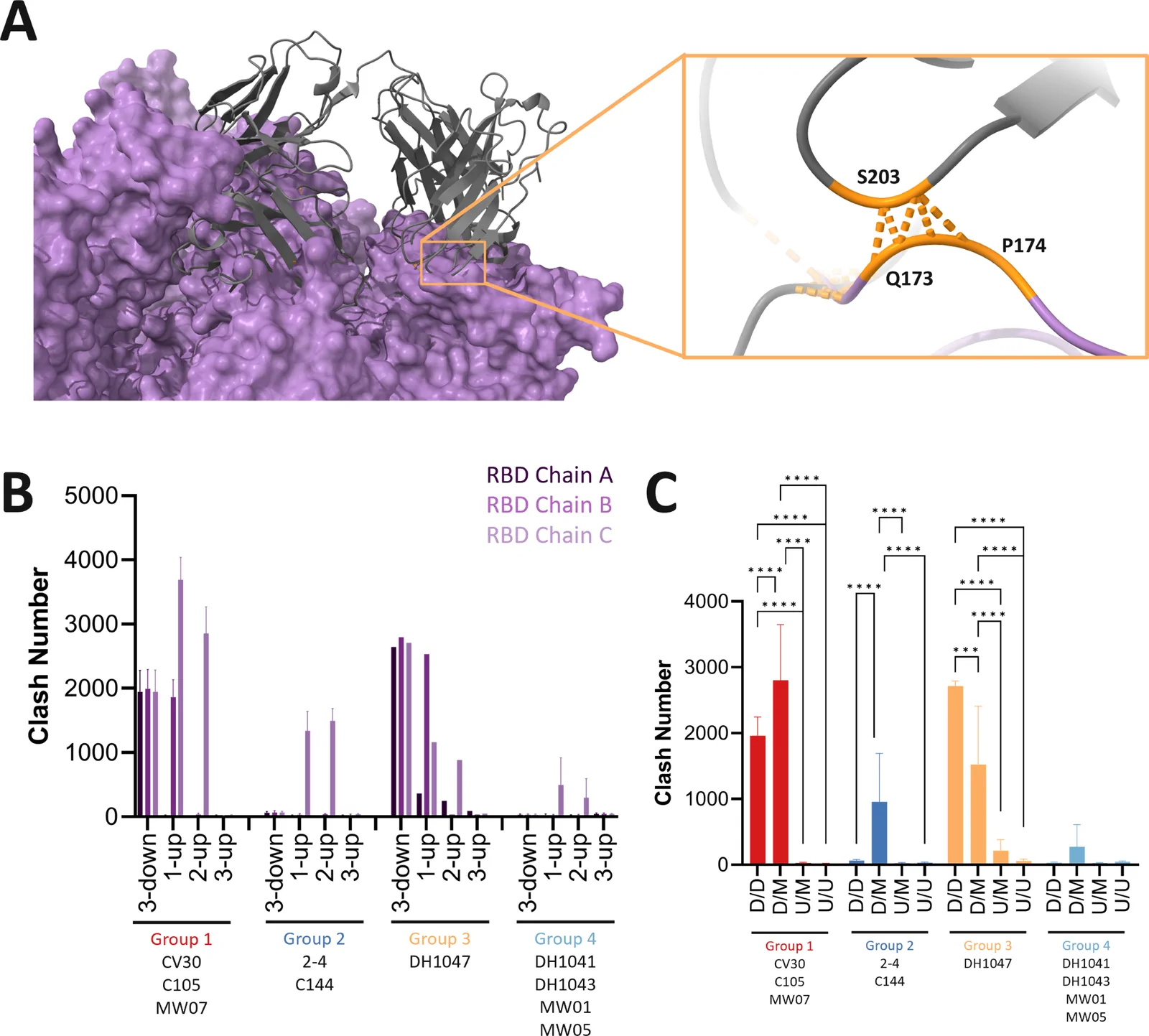
Quantified steric clashes for anti-SARS-CoV-2 antibody binding support group conformational regulation characteristics. (**A**) Representative images showing identification of clashes (orange) after antibody (gray, PDB: 6XCM [25]) alignment with spike trimer structure (purple, PDB: 7KRR [28]). Clashes defined as van der Waals overlap ≥1 Å after subtracting 0.40 Å for H-bonding. The right image shows a zoomed-in ribbon structure of the indicated section of the left image. (**B**) Graph of the average ± standard deviation of clashes for each antibody group in Table 1 binding to the RBD on each chain in 3-down, 1-up, 2-up, and 3-up trimer structures. In the 1-up trimer, Chain A is in the up conformation, and in the 2-up trimer, Chains A and B are in the up conformation. (**C**) Graph of average ± standard deviation of clashes for each antibody group in Table 1 when binding RBDs on down chains in 3-down trimer structures (D/D), down chains in mixed (1-up or 2-up) structures (D/M), up chains in mixed structures (U/M), or up chains in 3-up structures (U/U). *** and **** represent *P* ≤ 0.001 and *P* ≤ 0.0001, respectively, for a two-way ANOVA with post hoc Tukey’s test.

To investigate whether there was a structural basis for these differences in epitope and conformational regulation ability among the antibody groups, we aligned each antibody to baseline spike structures representing the four possible trimer states (i.e., 3-RBD-down, 1-up, 2-up, and 3-up). After aligning the antibodies to the RBD on each chain on the four spike structures, we quantified the resulting steric clashes for each antibody-RBD alignment, averaging the number of clashes when binding each chain for all antibodies in each group (Fig. 2A and B). We interpreted a large number of clashes (>1,000) as a large overlap that would prohibit binding, but a moderate number (~100) as potentially able to be accommodated with changes in the angle of the RBDs. For each antibody group with more than one antibody, we calculated the relative standard deviation of clashes for the group binding each chain/structure combination and found the average relative standard deviation within each group to be 31 ± 21%. Considering that the difference between the highest (3,904) and lowest (6) number of clashes for these alignments was nearly three orders of magnitude, we concluded that the number of clashes for each chain of each structure was quite consistent between antibodies of the same group.

We then used these data to investigate whether the four antibody groups showed distinct patterns of clashes when binding up vs down RBDs in all-down, all-up, or mixed (1-up or 2-up) trimer structures (Fig. 2C). The Group 1 (up-exclusive, no AME) and Group 3 (up-exclusive, FcγRI and FcγRIIb AME) antibodies had significantly more clashes when binding down RBDs in either all-down (D/D) or mixed (D/M) structures than when binding up RBDs in all-up (U/U) or mixed (U/M) structures. These large numbers of clashes would require antibodies from both groups to wait for unbound RBDs to transition from down to up and then stabilize this up conformation, shifting the equilibrium of trimers toward the 3-up state that lacks clashes. Notably, the Group 3 antibody showed over 10-fold more clashes than the Group 1 antibodies did when bound to up RBDs in mixed structures (Fig. 2C). These clash data therefore further support cryo-EM data showing up-exclusive binding for Groups 1 and 3, but also structural differences in binding for the two groups.

Interestingly, Group 2 (down-exclusive) and Group 4 (FcγRIIb AME) antibodies had remarkably similar profiles of clashes across the four spike structures (Fig. 2C), which is unexpected because these two antibody groups are nearly complete opposites in conformational regulation and AME characteristics (Table 1). Both groups had a small number of clashes except when binding down RBDs in mixed structures. For Group 2 antibodies, the thermodynamic unfavorability of the up conformation and the addition of favorable antibody interactions with an adjacent down RBD (19) are likely to trap the spike structures in the 3-down instead of 3-up state, consistent with cryo-EM structural data (19, 24). Group 4 antibodies lack these favorable interactions with adjacent down RBDs and had fewer clashes in mixed structures, suggesting that the existing clashes could be accommodated by small shifts in the angle of the neighboring up RBD. These shifts may create favorable interactions with adjacent up RBDs, which are observed in cryo-EM studies with Group 4 antibodies (9). These favorable interactions may allow the antibody to partially activate the spike to the mixed or all-up RBD states. However, additional analyses would be necessary to understand why Group 4 antibodies are up-preferring. This result demonstrates that the presence of steric clashing can predict some disallowed structures, but predicting which structures dominate in the presence of antibodies would likely require a structural analysis that allows for flexibility and additional favorable interactions.

Overall, quantifying these steric clashes allowed us to investigate conformational characteristics for antibodies that did not have conformational data through a method such as cryo-EM, expanding the information obtained from crystal structure data of antibody fragments bound to the isolated RBDs. This data revealed significant differences in binding to RBDs in different trimer structures between each antibody group, supporting the notion that epitope and conformation are predictive of antibodies that exhibit AME activity.

### Neutralization of entry into ACE2-expressing cells

**Fig 3:**
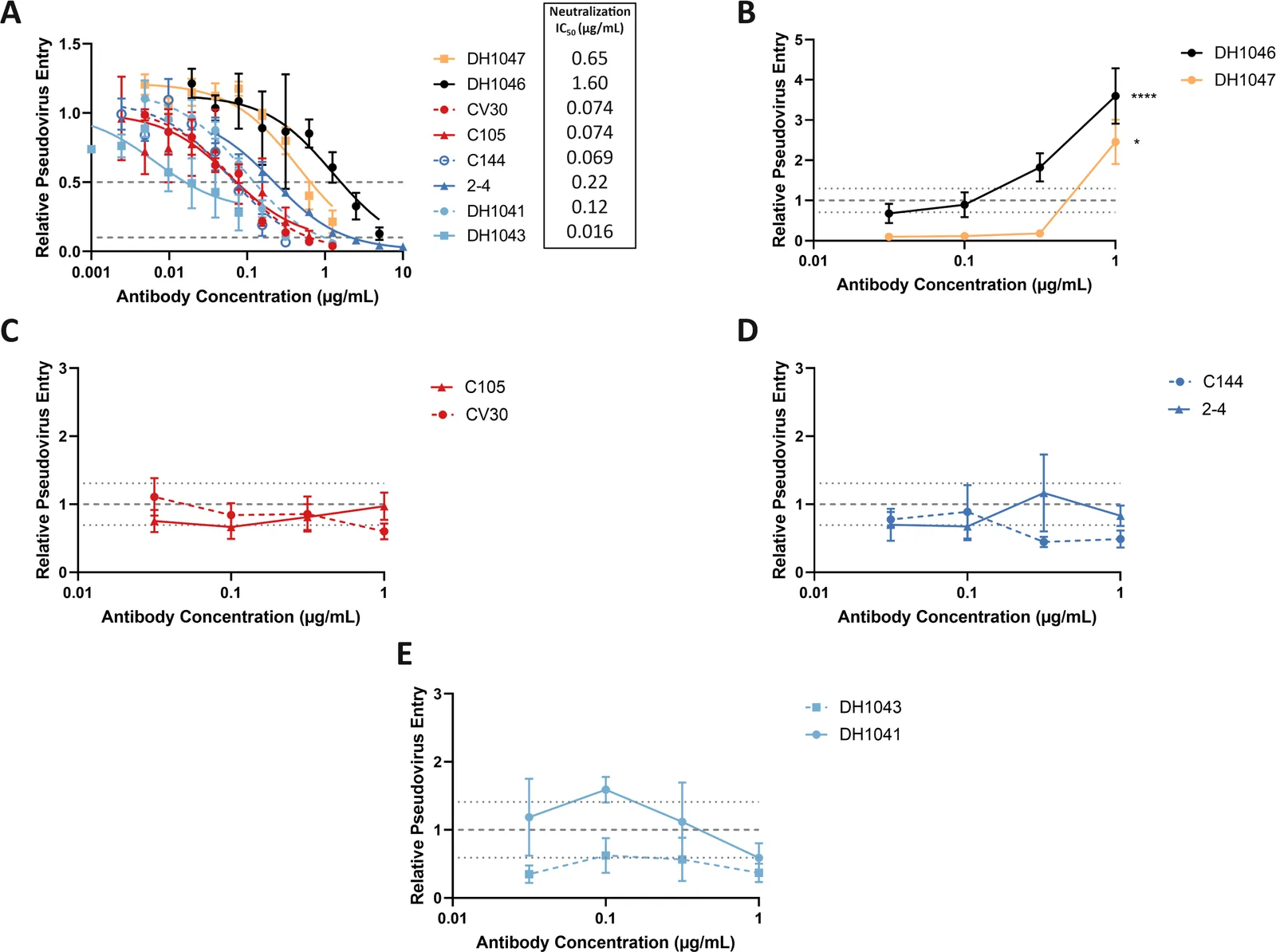
Anti-SARS-CoV-2 neutralizing antibodies of different groups have varying ability to mediate entry into THP1-derived macrophages. Entry of SARS-CoV-2-spike-pseudotyped viral particles in the presence of varying concentrations of antibody, normalized to entry of virus alone. (**A**) Neutralization of entry of pseudoviral particles into 293T-ACE2 HEK cells by mAbs. Lines for each antibody represent three-parameter nonlinear regression fit performed in Prism. Gray lines represent 50% (top) and 90% (bottom) inhibition of viral entry, respectively, with no mAb. Neutralizing IC_50_ for each antibody is also shown. (**B–E**) Sample data showing average ± SEM AME into THP1-derived macrophages for four to six technical replicates each. * represents *P* ≤ 0.05 and **** represents *P* ≤ 0.0001 for two-way ANOVA with post hoc Dunnett’s test comparing entry with each antibody concentration to the virus-only (no antibody) condition. Presence or absence of AME shown for each antibody was repeated in all biological replicates. Dashed and dotted gray lines represent average entry ± SEM, respectively, for virus alone. All of the three to four biological replicates with each antibody performed on different dates confirmed that DH1046 and DH1047 showed statistically significant entry, while the other six antibodies studied did not.

A subset of the antibodies described in Table 1 was then tested for their ability to neutralize SARS-CoV-2-spike-pseudotyped (Wuhan-Hu-1 strain with D614G mutation) lentiviral particle entry into HEK 293T-ACE2 cells to directly compare neutralization using the same pseudovirus-cell system. Consistent with previous studies, all antibodies we tested neutralized pseudovirus entry into the 293T-ACE2 cells (Fig. 3A). Notably, the neutralization IC_50_s of DH1046 (1.60 μg/mL) and DH1047 (0.65 μg/mL), the Group 3 FcγRI and FcγRIIb AME antibodies, were both higher than the IC_50_s of each of the Group 4 FcγRIIb-only AME antibodies (0.0074–0.22 μg/mL), which also supports previously published neutralization data.

### Antibody-mediated entry into THP1-derived macrophages

Many of the antibodies described in Table 1, particularly in Groups 1 and 2, had not previously been tested for AME, and those that had were largely tested in cells engineered to express FcγRs (9). To compare AME into immune cells naturally expressing FcγRI, the FcγR shown to be clinically relevant for AME (7), using a shared pseudovirus-cell system, we tested a subset of the mAbs in Table 1 (those available due to NIH funding), for their ability to mediate pseudoviral entry into THP1-derived macrophages (Fig. 3B through E). Both Group 3 antibodies, DH1047 and DH1046, mediated statistically significant entry of the spike pseudovirus into THP1-derived macrophages compared to the same-day no antibody condition (Fig. 3B). These results therefore support previous work showing AME by DH1047 and DH1046 mAbs into FcγRI-expressing TZM-bl cells (9), and are expected given that THP1-derived macrophages have been shown to express FcγRI (29). To our knowledge, none of the Group 1 or Group 2 antibodies we examined had previously been tested for AME ability. We found that none of them showed any significant AME into the THP1-derived macrophages (Fig. 3C and D), which allowed us to update Table 1 with additional information about these antibodies (Table S1). Similarly, neither DH1043 nor DH1041 (Group 4) mediated significant entry into the THP1-derived macrophages (Fig. 3E). Notably, this lack of AME for Group 4 mAbs is expected given prior work showing that they only mediate entry via FcγRIIb (9), which is minimally expressed on THP1-derived macrophages (29). Due to day-to-day variability in AME signal, sample data from a single biological replicate are shown for each antibody in Fig. 3, but all biological replicates of these AME experiments showed the same results (Fig. S1), with statistically significant AME shown only for DH1046 and DH1047.

### Inhibition of AME by up-exclusive and down-exclusive antibodies

**Fig 4:**
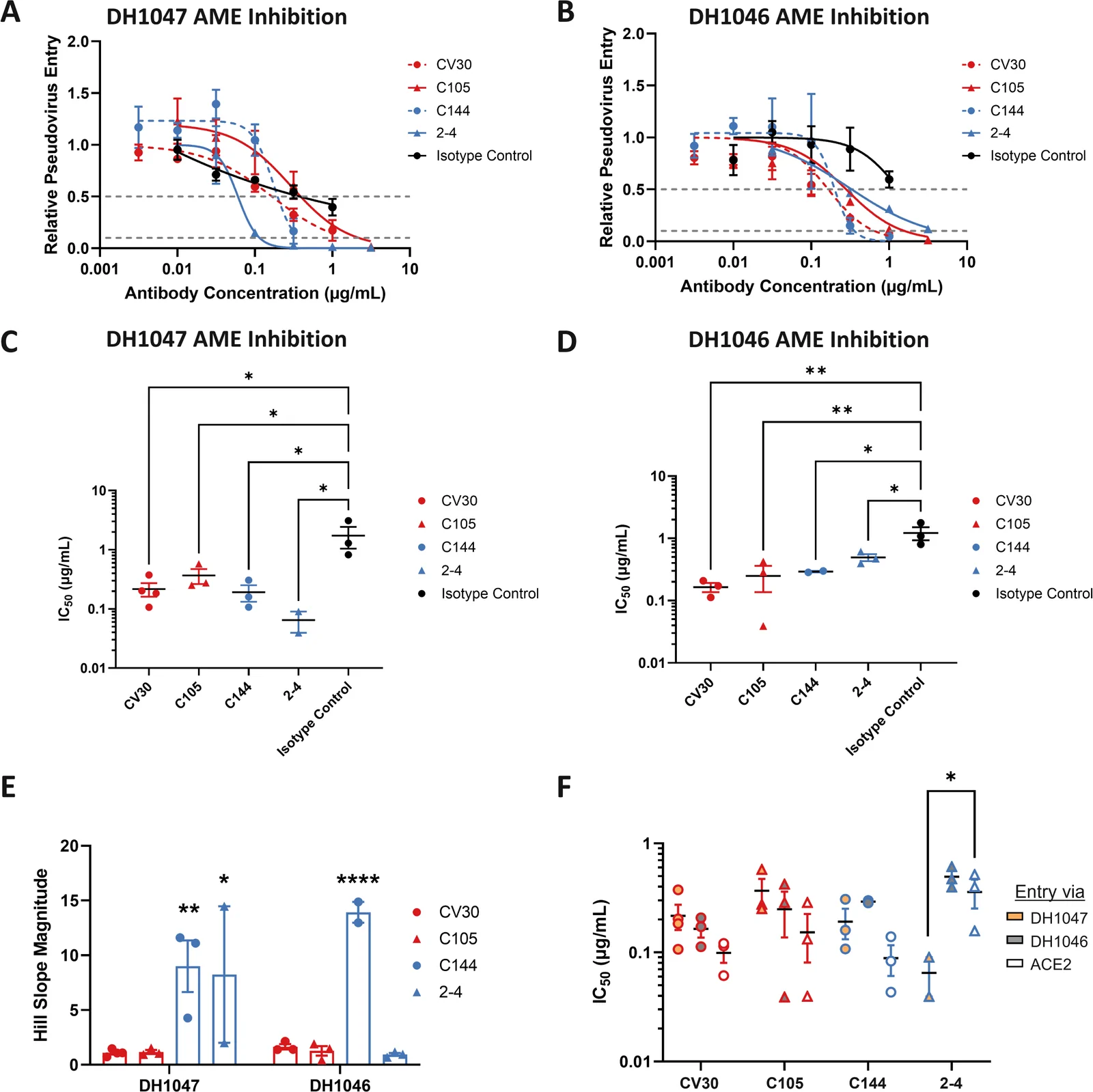
Up-exclusive (Group 1) and down-exclusive (Group 2) antibodies neutralize DH1047- and DH1046-mediated SARS-CoV-2 entry into THP1-derived macrophages. Entry of SARS-CoV-2-spike-pseudotyped pseudoviral particles in the presence of 1 μg/mL DH1047 (**A–C**) or DH1046 (**B–D**) and varying concentrations of inhibitory antibodies or isotype control, normalized relative to pseudovirus with DH1047 or DH1046 only. (**A and B**) Gray lines represent 50% (top) and 90% (bottom) inhibition, respectively. Points represent the average ± SEM of three biological replicates (for (**A**), CV30 is four replicates, 2-4 is two replicates). Lines for each antibody represent nonlinear regression three-parameter Hill model with a fixed 0 baseline performed in Prism. (**C and D**) IC_50_ for each antibody based on nonlinear regression fits shown in panels **A and B** for each biological replicate; lines represent the average ± SEM of the shown points. *P* values shown for ordinary one-way ANOVA with post hoc Dunnett’s test. (**E**) Hill slopes from nonlinear fits in panels **A and B** for each biological replicate; bars represent the average ± SEM of the shown points. *P* values shown for two-way ANOVA with post hoc Tukey’s test. (**F**) IC_50_s for neutralization of entry via DH1047 AME, DH1046 AME, and ACE2 for each antibody. Lines and bars represent the average ± SEM for the shown biological replicates. IC_50_s calculated using nonlinear fits shown in corresponding graphs in Fig. 3 and this figure. *P* values were calculated with for two-way ANOVA with post hoc Dunnett’s test, and indicated by *P ≤ 0.05, **P ≤ 0.01, and ****P ≤ 0.0001.

To determine whether individual antibodies are able to inhibit AME into THP1-derived macrophages, we tested the Group 1 (up-exclusive) and Group 2 (down-exclusive) antibodies for their ability to inhibit entry mediated by the Group 3 antibodies. All the tested Group 1 and Group 2 antibodies were able to inhibit AME by both Group 3 antibodies in a concentration-dependent manner (Fig. 4A and B). To determine whether the antibodies were inhibiting AME by competitively binding the FcγRs, we also tested the inhibitory ability of an isotype control antibody. This isotype control failed to reach 50% inhibition of AME for both Group 3 antibodies in the equivalent tested concentrations. Additionally, the isotype control had a significantly higher IC_50_ for AME than all of the Group 1 and Group 2 inhibitory antibodies when fitting the inhibition data using a nonlinear regression Hill model with a fixed 0 baseline (Fig. 4C and D). Overall, these data suggest that both Group 1 and Group 2 antibodies can inhibit entry mediated by both Group 3 antibodies, and likely do so through interaction with the SARS-CoV-2 spike protein, rather than exclusively by competing to bind the FcγRs.

We then investigated whether antibodies with varying conformational regulation and epitope differentially inhibit AME. From the nonlinear regression model fits, we found that the magnitudes of Hill coefficients for the Group 1 (up-exclusive) antibodies did not differ significantly from 1 when inhibiting AME by either Group 3 antibody (Fig. 4E). Therefore, the Group 1 antibodies demonstrate no cooperativity when inhibiting entry by these antibodies. In contrast, the magnitudes of Hill coefficients for Group 2 (down-exclusive) antibodies inhibiting AME by Group 3 mAb DH1047 were significantly greater than 1, indicating positive cooperativity for both. When inhibiting AME by Group 3 mAb DH1046, the Hill coefficient magnitude for Group 2 antibody C144 remained significantly greater than 1, indicating the same positive cooperativity shown for C144 inhibiting DH1047 AME. In contrast, Group 2 antibody 2-4 had a Hill coefficient magnitude that did not significantly differ from 1 when inhibiting DH1046 AME, indicating lack of cooperativity. Collectively, these results suggest that when inhibiting AME by Group 3 antibodies, Group 1 antibodies consistently have noncooperative inhibition, but Group 2 antibodies often show positive cooperativity. As shown in Fig. 4A and B, this positive cooperativity allows these Group 2 antibodies to inhibit nearly all AME at concentrations barely above IC_50_.

We also compared the IC_50_s for Group 3 AME inhibition of each antibody to its IC_50_ for neutralization of ACE2-mediated entry (Fig. 4F). Group 1 antibodies CV30 and C105 and Group 2 antibody C144 all had AME inhibition IC_50_s that did not differ significantly from their respective neutralization IC_50_s. Group 2 antibody 2-4 had a significantly lower AME inhibition IC_50_ than neutralization IC_50_ when inhibiting AME by DH1047, but not when inhibiting AME by DH1046. Relatedly, 2-4 is the only antibody that was more effective (almost fourfold) at inhibiting Group 3 AME than at neutralizing ACE2-mediated entry when comparing IC_50_s. As a result, 2-4 was the most effective inhibitor of DH1047 AME (IC_50_: 0.059 μg/mL) and the least effective inhibitor of DH1046 AME (IC_50_: 0.29 μg/mL) (Fig. 4C and D). This nearly fivefold lower effectiveness at inhibiting DH1046 vs DH1047 AME was notably greater in magnitude than the differences in AME IC_50_ for the other three inhibitory antibodies, which all differed by under twofold. Overall, all tested up-exclusive (Group 1) and down-exclusive (Group 2) antibodies were able to inhibit entry mediated by two different Group 3 mAbs at neutralizing concentrations or lower.

## DISCUSSION

Previous work has identified epitope regions on the SARS-CoV-2 RBD and associated them with up-exclusive and down-exclusive binding (30, 31). Additionally, the ability to mediate SARS-CoV-2 entry via FcγRIIb has been previously shown to correlate with epitope (12). Hastie et al. used competition assays to define seven anti-SARS-CoV-2 antibody communities, subsequently finding that antibodies in each community bind a similar region on the RBD distinct from those in other communities (30). In this work, we grouped antibodies by conformational regulation and AME and similarly found that antibodies within groups targeted similar epitopes distinct from those of other groups. We thereby identified the locations of unique epitopes associated with AME and determined that only up-preferring and up-exclusive but not down-exclusive antibodies mediate entry into FcγRI-expressing cells. To our knowledge, these epitope locations and conformational regulation characteristics have not been previously identified as unique features of AME antibodies. In addition, the structural epitope and clash analyses we performed suggest not only that certain antibody epitopes and clash patterns are associated with AME in general, but also that antibodies mediating entry via different FcγRs target unique epitopes on the RBD and create unique clash patterns that predict conformational preferences. We thereby defined four groups of anti-SARS-CoV-2 antibodies with unique AME, conformational regulation, and epitopes, suggesting that these characteristics are both connected and predictive.

**Fig 5:**
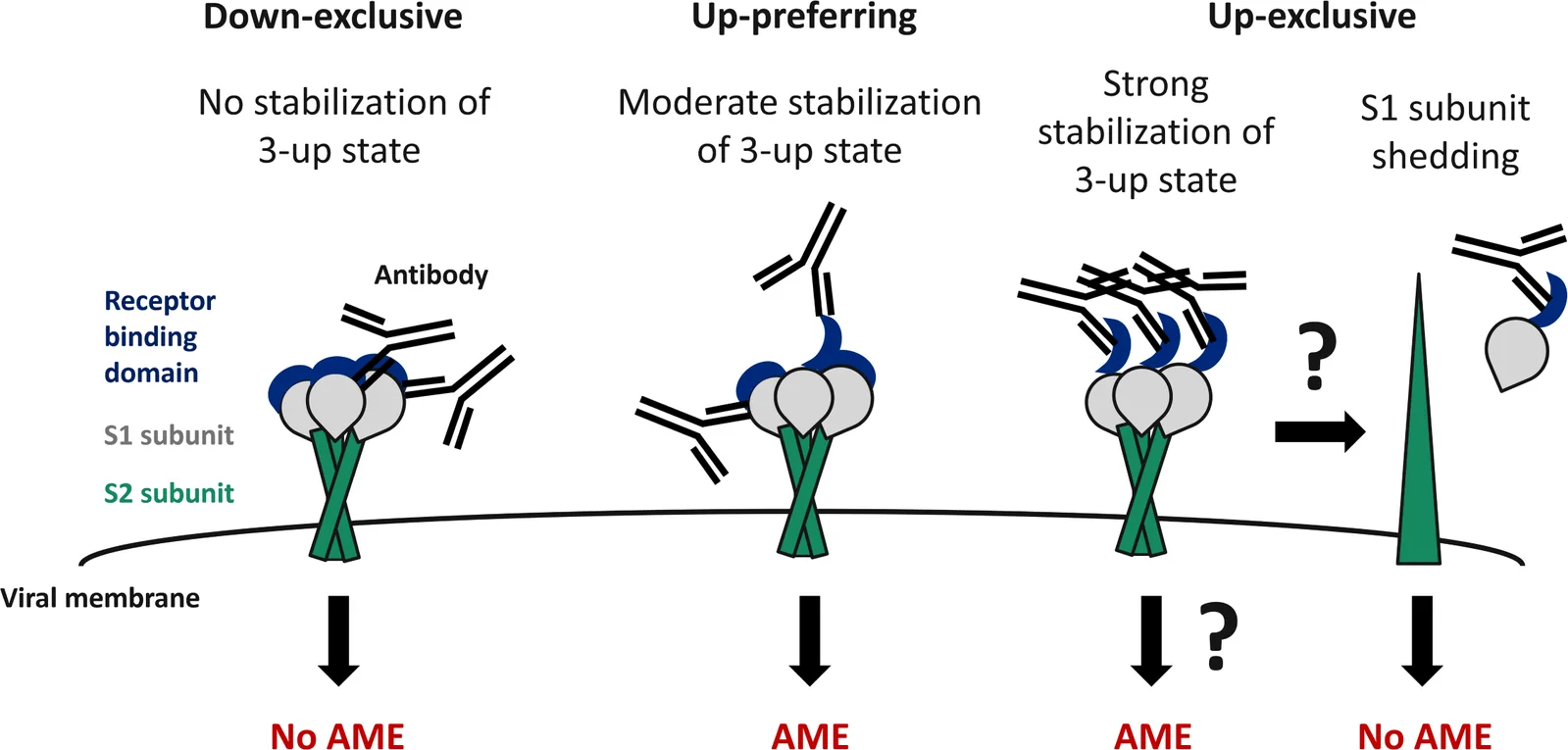
Proposed relationship between SARS-CoV-2 RBD conformational dynamics and antibody-mediated entry (AME).

It has been previously shown that viral fusion is required for AME (10). The lack of AME that we measured for down-exclusive antibodies suggests that the requirement of the up RBD conformation to initiate fusion with ACE2-expressing cells (23) may be similarly required for fusion with FcγR-expressing cells during AME. Therefore, as illustrated in Fig. 5, antibodies may need to allow, or even promote, the up RBD conformation to mediate FcγR-dependent entry. Notably, while up-exclusive antibodies promote the 3-RBD-up state, prior work indicates that by strongly promoting the 3-up state, up-exclusive antibodies that mimic ACE2 binding (Group 1) can enable premature cleavage and irreversible shedding of the RBD-containing S1 subunit from the virus (21). It seems likely that this irreversible S1 subunit shedding could be occurring prior to viral binding, thereby preventing Group 1 up-exclusive antibodies from mediating entry, as reflected in this work. However, one Group 3 antibody that is up-exclusive, but binds a different epitope than ACE2 does, mediated AME in our assay, and prior work shows AME for up-preferring Group 4 antibodies (9). It remains an open question why up-preferring antibodies and some up-exclusive antibodies mediate AME. To our knowledge, no AME antibodies from Group 3 or Group 4 have been tested for premature S1 subunit shedding. Additional testing could elucidate whether binding angle, weaker promotion of the up RBD by up-preferring antibodies, bivalent binding shown for some AME antibodies (10), or additional interactions with adjacent up RBDs shown for one Group 3 antibody (9) impact how these antibodies affect spike conformational regulation and AME. These changes could allow the antibodies time to mediate binding of the antibody-virus complex to the FcγR-expressing cells and initiate subsequent AME without inducing premature S1 subunit shedding. Overall, our work indicates a correlation between conformational regulation and AME abilities of antibodies, which may contribute to why some, but not all, antibodies mediate FcγR-dependent entry.

We also found that neutralizing antibodies can inhibit SARS-CoV-2 macrophage entry mediated by both Group 3 AME antibodies we studied. Both up-exclusive (Group 1) and down-exclusive (Group 2) antibodies inhibited AME more effectively than did an isotype control that can act as a competitor for FcγRs. This finding suggests that AME inhibition by up-exclusive and down-exclusive antibodies is not only due to competitive inhibition of the FcγR but involves interaction with the SARS-CoV-2 spike, potentially through inhibition of spike binding and conformational regulation preventing viral fusion.

Notably, Group 1 antibodies overlap in epitope and therefore likely compete with the Group 3 AME antibody DH1047, but they may also inhibit AME conformationally by inducing S1 subunit shedding, as described above. In contrast, neither Group 2 antibody epitope overlaps at all with that of DH1047. This lack of overlap suggests that the Group 2 antibodies likely inhibit Group 3 AME exclusively noncompetitively, through conformational regulation. This finding subsequently raises the question of whether the down-exclusive binding of these antibodies is related to the positive cooperativity seen for Group 2, but not Group 1, inhibition of Group 3 AME. Group 2 antibodies have been shown to stabilize its primary bound RBD and an adjacent down RBD (19), which would likely offer cooperativity for subsequent down-exclusive antibodies to bind the adjacent RBD and inhibit the up conformation that is required for viral fusion (23) and AME (10). In particular for DH1047, which is known to bind largely the inner face buried in the down conformation (9), stabilizing down RBDs would likely directly, but noncompetitively, impede DH1047 binding and subsequent AME. Why Group 2 antibody 2-4 loses this cooperative behavior when inhibiting DH1046 AME remains unclear without structural data of DH1046 binding. Nevertheless, in this work, we found that differences in RBD conformational regulation by antibodies are associated with differences in the use of competitive and noncompetitive mechanisms and the role of cooperativity when inhibiting AME. Notably, it has been found that noncompetitive conformational inhibitors are typically more effective *in vivo* than competitive inhibitors (32, 33).

The structural analysis and the correlations between antibody epitope, AME, and AME inhibition, and conformational regulation described in this work relied heavily on previously published structural data. These structures were determined by relatively low-throughput methods such as cryo-EM and x-ray crystallography, subsequently limiting the number of antibodies with known conformational regulation and epitope. In particular, structural data for additional Group 3 antibodies would be needed to elucidate the relationship between conformational regulation, epitope, and AME. AME testing may also be warranted for antibodies with existing structural data showing a high degree of epitope overlap with Group 3 antibody DH1047, such as S2X259 (22/25 epitope residues overlap with DH1047 epitope of 37 residues) (34, 35), CoVIC-063 (visual inspection) (30), and C135 (3/15 epitope residues overlap with DH1047) (19, 36). Notably, DH1047 and some of these highly overlapping antibodies have been attributed to different RBD groups (35), demonstrating that existing RBD groupings may not alone be predictive of AME. Wider use of existing ELISA-type assays (37) to test for differences in RBD conformational regulation would also allow for higher throughput classification of antibodies by this characteristic. Additionally, development and use of epitope libraries (38) would allow for higher throughput identification of functional, not just structural, epitopes of anti-SARS-CoV-2 antibodies to further define shared group epitopes. Additional data and methods could further elucidate the relationship between conformational regulation, epitope, and AME.

Several limitations affect the conclusions drawn from this work. Only one to three antibodies per group had known conformational regulation, epitope, and AME ability. Therefore, the classifications of these groups, and observed similarities and differences within and between them in our study may not represent every antibody with similar characteristics. Due to the limited conformational data available, IgG subclasses and Fc glycosylation of the antibodies used were not controlled for, and diversity in these could affect AME and AME inhibition through differences in FcγR engagement. Additionally, our work focused on a small sample of monoclonal antibodies. How the diversity of antibodies and epitopes in polyclonal sera would affect the neutralization, AME, and AME inhibition of these antibodies is unclear. Another limitation is that our epitope and clash analyses were limited by the static nature of crystal and cryo-EM structures, the limited detection of quaternary spike interactions when using only Fab fragments, and the lack of representation of glycan heterogeneity. In addition, our AME and AME inhibition assays did not include mechanistic controls for fusion blockade or Fc receptor blockade, though notably, prior work shows Fc-receptor dependence of the AME antibodies tested (9). While our work supports understanding of AME and AME inhibition into macrophages using an *in vitro* monocyte-derived macrophage cell line assay, additional studies using primary macrophages, which have been implicated in COVID-19 inflammation (7), and may have different FcγR profiles, would further elucidate the physiological relevance of these antibody effects. Finally, the Wuhan-Hu-1 spike variant was used in this work because the antibodies tested were elicited to target this variant. Additional AME studies using virus pseudotyped with more recent spike variants would provide more insight into how binding, neutralization, and AME differ when targeting different SARS-CoV-2 variants. Notably, however, the mutations associated with a more recent Omicron variant were spread among the epitopes of all antibody groups defined in this work, not clearly favoring or disfavoring AME antibody binding.

Another caveat of this work is that the correlation between AME *in vitro* and clinical outcomes remains unclear. Notably, a study on AME antibodies showed little enhancement of lung pathology 4 days after SARS-CoV-2 challenge in non-human primates (9). However, that study used AME antibodies as a prophylaxis, where the neutralizing effect could prevent a high viral load. It remains unclear whether AME may have an impact on pathology when antibodies are introduced when viral load is high, so that the effect could be greater. This question is relevant to the administration of antibodies as a therapeutic at the peak of infection and to breakthrough infections that occur when immunity from prior infection or vaccination has faded.

Existing vaccines aim to elicit neutralizing RBD antibodies, of which most AME antibodies are a subset (9, 10). Importantly, vaccines were shown not to elicit AME activity in uninfected patients (7) and continue to be protective against long COVID-19 (39) and severe acute COVID-19 (40). Therefore, understanding the connection between epitope, conformational regulation, and AME explored in this work could allow us to further improve vaccine antigen and therapeutic design for COVID-19 and related viral diseases. Notably, we found that the FcγRI AME antibodies were also the most weakly neutralizing of ACE2-mediated entry, suggesting that avoiding induction of AME antibodies may not decrease the availability of neutralizing antibodies. Our AME inhibition results also suggest that the most strongly neutralizing antibodies may also inhibit AME effectively, but further testing using varying antibody concentrations and combinations, including human serum, would further elucidate the physiological relevance of these results. Designing vaccine antigens that both avoid AME antibodies and promote AME inhibitory antibodies could add functionality to these vaccines that are already effective, allowing them to simultaneously neutralize ACE2 entry to decrease viral load and prevent FcγR-dependent AME to minimize inflammation.

## MATERIALS AND METHODS

### Epitope analysis

Structures for CV30 (PDB: 6XE1) (21), C105 (6XCM) (25), MW07 (7DK2) (10), DH1047 (7LD1) (9), DH1041 (7LAA) (9), DH1043 (7LJR) (9), MW01 (7DJZ) (10), MW05 (7DK0) (10), 2-4 (6XEY) (12), C144 (7K90) (19), and ACE2 (6M0J) (41) were all obtained from the Protein Data Bank (PDB). Antibodies that were found in their respective structures to bind only up or down RBDs were defined as “up-exclusive” and “down-exclusive,” respectively. Antibodies that caused a shift in the trimer population away from the 3-down state but were not found to require the up state to bind were classified as “up-preferring.” Some antibodies were classified as having “unknown” conformational regulation if the regulation of spike trimer conformations by that antibody was not clear. SARS-CoV-2 spike epitopes for antibody Fab fragments and ACE2 were defined as residues within 6 Å of the Fab or ACE2 and calculated using Pymol. Images of shared epitope residues on the RBD were generated using Chimera.

### Clash analysis

Alignment of the original antibody structures to the clash analysis structures was performed using Pymol. Four clash analysis structures of SARS-CoV-2 spike with a D614G mutation were identified in the literature that represent the four possible spike trimer states: 3-down (PDB: 7KRQ) (28), 1-up (PDB: 7KRR) (28), 2-up (PDB: 7BNO) (42), and 3-up (PDB: 7WO5) (43). For each of the three chains (spike protomers) in each of these clash analysis structures, the RBD (residues 333-527) (44) was aligned to the RBD of the corresponding chain on the original antibody structure. In these structures, the RBD in Chain A is up in the 1-up trimer state, and the RBDs in Chains A and B are up in the 2-up trimer state. For original antibody structures that contained fewer than three RBDs, as many RBDs as available were used throughout the analysis, but RBDs were aligned with multiple chains of the clash analysis structures as necessary. A new PDB containing only the clash analysis structure and the single aligned Fab fragment was thereby generated for each Fab/chain/trimer state combination. For each new PDB, clashes were defined as van der Waals overlap ≥1 Å after subtracting 0.40 Å for H-bonding and quantified using Chimera. Relative standard deviation was calculated by dividing the standard deviation of all antibodies in the associated group binding each chain/structure combination by the mean number of clashes when binding that chain/structure combination. For example, the three Group 1 antibodies binding Chain A in the 3-down structure had 1,957, 1,612, and 2,278 clashes. The standard deviation (333) divided by the mean (1,949) of those numbers produces a relative standard deviation of 0.17 for that antibody group/structure/chain combination.

### Production of SARS-CoV-2-spike-pseudotyped lentiviral particles

Non-replicative HIV-1-derived lentiviral particles pseudotyped with SARS-CoV-2 spike were produced as previously described (45). In brief, human embryonic kidney (HEK) 293T cells were co-transfected with five plasmids: one containing the lentiviral backbone and reporter proteins (BEI catalog number NR-52516), three for key HIV-1 proteins (Tat [NR-52518], Gag-Pol [NR-52517], and Rev [NR-52519]), and one encoding the entry protein. To produce SARS-CoV-2-spike-pseudotyped viral particles, the entry protein plasmid that was used encoded a SARS-CoV-2 Wuhan-Hu-1 spike variant containing a C-terminal 21-amino-acid deletion and a D614G mutation (NR-53765). For positive and negative control pseudoviruses, an HDM-VSVG plasmid encoding VSV G and a transfection carrier plasmid (Promega), respectively, were instead used as the entry protein plasmid. Transfections were performed using Lipofectamine 2000 (Invitrogen) according to the manufacturer’s protocols. All resulting lentiviruses contained luciferase expressed with a CMV primary promoter and fluorescent ZsGreen expressed with an internal ribosome entry site (IRES). Following a 72-h transfection using Lipofectamine, the supernatant was filtered through a 0.45 µm SFCA low-protein-binding filter to harvest the pseudoviruses. Prior to transfection, HEK cells were cultured in Dulbecco’s modified Eagle medium (DMEM, Gibco) with 10% heat-inactivated FBS (R&D Systems), 100 U/mL Pen-Strep (Gibco), and 2 mM l-glutamine (Gibco). For the transfection, cells were switched to RPMI (Gibco) with 10% heat-inactivated FBS (R&D Systems), 100 U/mL Pen-Strep (Gibco), 1 mM sodium pyruvate (Gibco), and 2 mM l-glutamine (Gibco). All cell culture and experiment incubation steps described in this work were performed at 37°C with 5% CO_2_.

### Measuring antibody-mediated neutralization of pseudovirus entry into ACE2-expressing cells

Measurement of viral entry into ACE2-expressing cells was measured as previously described (45). Briefly, tissue-culture-treated 96-well plates were seeded with either 293T or 293T-ACE2 cells (~25,000 cells/well) approximately 16 h before infection with pseudovirus. For all pseudovirus infections described in this work, pseudovirus was added at a 2:3 dilution in fresh media, with a total infection volume of 150 μL per well. Pseudoviruses were preincubated with varying concentrations of mAbs for 1 h before being added to the seeded cells and allowed to infect the cells for 50 h. Three wells for each antibody concentration were used in each biological replicate. Following the infection period, pseudovirus-containing medium in each well was replaced with 100 µL of fresh phenol-red-free DMEM (Gibco) with 2 mM DMNPE-caged luciferin (GoldBio). Luminescence measurements were taken as frequently as possible over approximately 5 min following the addition of luciferin. A decrease in luminescence in this assay indicates neutralization. For the incubation steps described in this section, cells and pseudoviruses were cultured and incubated at 37°C with 5% CO_2_. The medium used for this section was DMEM with 10% heat-inactivated FBS (R&D Systems), 100 U/mL Pen-Strep (Gibco), and 2 mM l-glutamine (Gibco). Data for each antibody were fit to a nonlinear regression (inhibitor) vs response model with three parameters and graphed using GraphPad Prism. These fits were used to calculate neutralization IC_50_, defined as the concentration required to reach 50% inhibition of the entry with no antibody, which was calculated for each biological replicate.

### Measuring antibody-mediated pseudovirus entry into FcγR-expressing cells

In order to measure antibody-mediated pseudoviral entry into macrophages, suspended THP-1 monocytes were differentiated into adherent macrophages using a method modified from one previously described (29). Briefly, 300 μL cells at 600,000 cells/mL with 7.5% phorbol-12-myristate 13-acetate (PMA) were added per well to tissue-culture-treated, white-walled 96-well plates (Corning) and incubated for 24 h. Six wells were used for each antibody concentration per biological replicate. Following this PMA priming step, cells were washed once with DPBS before being rested with fresh media for 24 h prior to pseudoviral infection. The medium used for these cells was RPMI with 10% heat-inactivated FBS (R&D Systems), 100 U/mL Pen-Strep (Gibco), 1 mM sodium pyruvate (Gibco), and 2 mM l-glutamine (Gibco).

As described above for the neutralization assay, pseudoviruses were incubated with mAbs at varying concentrations for 1 h before being added to the FcγR-expressing macrophages. Following a 48-h infection incubation, pseudovirus entry into the cells was measured using luminescence, as described above. Luminescence readings were taken as often as possible over approximately 6 min following the addition of luciferin; readings at approximately 30 s, 2 min and 30 s, and 4 min and 30 s were averaged for each well on each day. Increase in luminescence for this assay indicates AME. Data were analyzed using a two-way ANOVA with a post hoc Dunnett’s test and graphed using GraphPad Prism.

### Inhibition of AME in THP1-derived macrophages

AME into THP1-derived macrophages was measured as described above, with a slight modification of adding a constant concentration of AME antibody to the pseudovirus, followed by varying concentrations of inhibitory antibody or isotype control (antibody targeting the SARS-CoV-2 nucleocapsid [N], which does not exist in the pseudoviral particles used in this work). Antibody-pseudovirus mixtures were then incubated for 1 h before infection and luminescence measurement as described above. Six wells were used for each antibody concentration per biological replicate. Anti-nucleocapsid mAb 1A6 (Invitrogen) was used as a control antibody to bind FcγRs but not the pseudoviruses, which did not contain nucleocapsid. To calculate relative entry, the background luminescence of spike pseudovirus alone was subtracted from each luminescence value before normalization to the average luminescence for the spike with AME antibody only positive control. Luminescence readings were taken as often as possible over approximately 6 min following the addition of luciferin; readings at approximately 0.5, 2.5, and 4.5 min were averaged for each well on each day. The medium used to culture the THP1 cell line and experiments was RPMI (Gibco) with 10% heat-inactivated FBS (R&D Systems), 100 U/mL Pen-Strep (Gibco), 1 mM sodium pyruvate (Gibco), and 2 mM l-glutamine (Gibco).

These data were fit to a nonlinear regression three-parameter Hill model with a fixed 0 baseline and graphed using GraphPad Prism. These fits were used to calculate AME IC_50_, defined as the concentration required to reach 50% inhibition of the entry with AME antibody alone, which was calculated for each biological replicate. Further analysis comparing each antibody’s AME IC_50_s to its respective neutralization IC_50_ or the isotype control IC_50_ was performed using a one-way and two-way ANOVA, respectively, with post hoc Dunnett’s test. Additional two-way ANOVAs to compare each antibody’s Hill slope to each other antibody’s Hill slope and to compare each Hill slope to −1 were performed using post hoc Tukey’s and Šidák’s tests, respectively. Hill slopes were taken from the nonlinear regression fits described above, and the ANOVAs were also performed using GraphPad Prism.

## SUPPLEMENTAL MATERIAL

Data set S1 spectrum.00120-26-s0001.xlsx Complete data sets for all figures.

Fig. S1 and Table S1 spectrum.00120-26-s0002.pdf Fig. S1: Biological replicate data showing AME into THP1-derived macrophages by DH1046 and DH1047. Table S1: Unique antibody epitopes are associated with different conformational and AME effects for SARS-CoV-2.

ASM does not own the copyrights to Supplemental Material that may be linked to, or accessed through, an article. The authors have granted ASM a non-exclusive, world-wide license to publish the Supplemental Material files. Please contact the corresponding author directly for reuse.
