# Supplementary material for: Antibody-mediated SARS-CoV-2 entry and conformational regulation
Source: Microbiol Spectr. 2026 Aug 21;14(10):e00120-26. doi: 10.1128/spectrum.00120-26 (PMC13689087; doi:10.1128/spectrum.00120-26)
Supplement: Supplementary file 1 [file spectrum.00120-26-s0002.pdf]

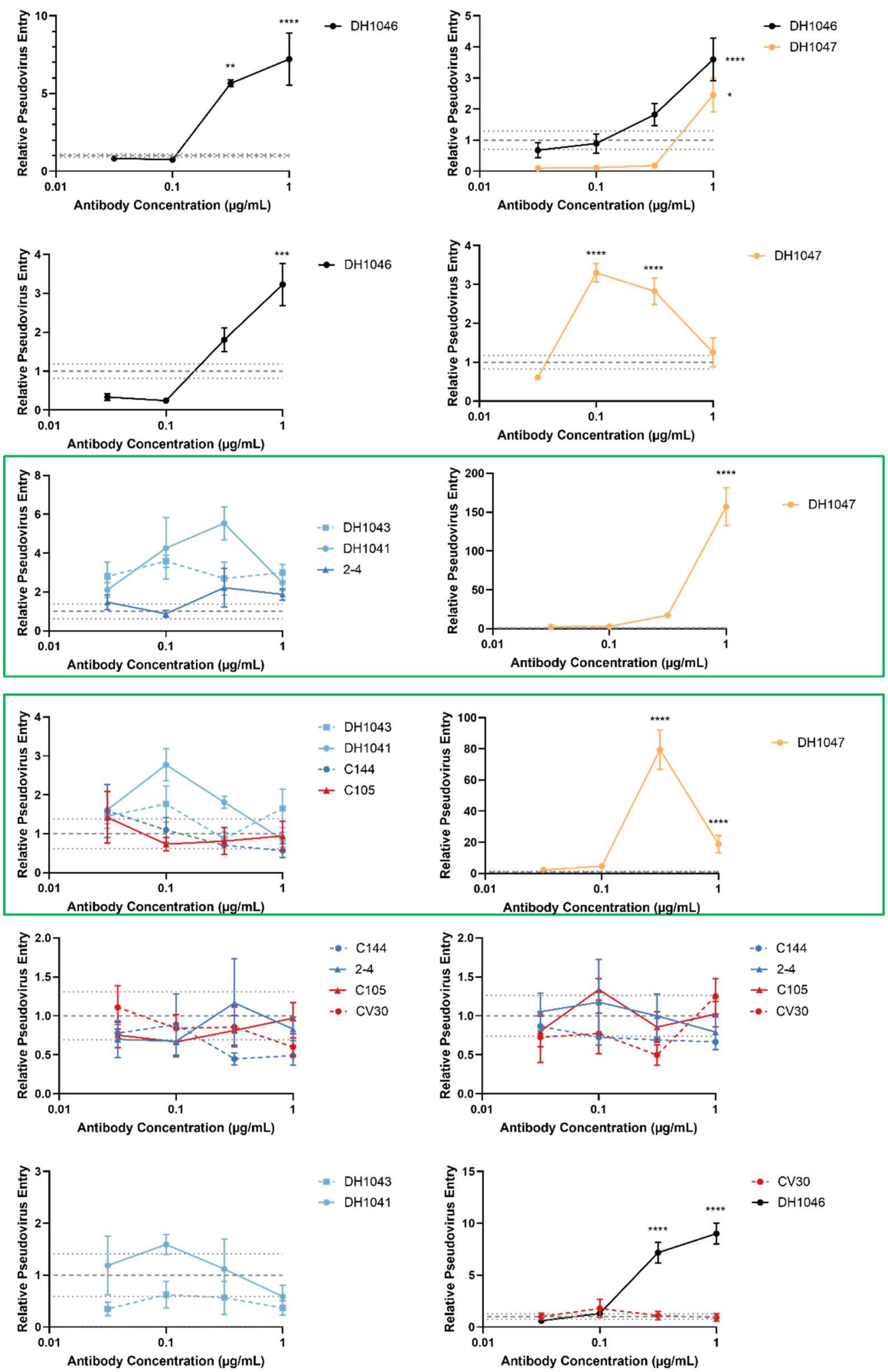

Figure S1. Biological replicate data showing AME into THP1-derived macrophages by DH1046 and DH1047. Entry of SARS-CoV-2-spike-pseudotyped viral particles in the presence of varying concentrations of antibody normalized to entry of virus alone. Points represent average ± SEM AME into THP1-derived macrophages for 4-6 technical replicates each. * represents $p \leq 0.05$, ** represents $p \leq 0.01$, *** represents $p \leq 0.001$, and **** represents $p \leq 0.0001$ for two-way ANOVA with post hoc Dunnett's test comparing entry with each antibody concentration to the virus only (no antibody) condition. Green boxes indicate data was collected in the same experiment. Dashed and dotted grey lines represent average entry ± SEM, respectively, for virus alone. Note that on each day, only DH1046 and DH1047 mediated statistically significant entry that was dependent on antibody concentration. Table S1. Updated version of Table 1 - Unique antibody epitopes are associated with different conformational and AME effects for SARS-CoV-2.

Table S1. Updated version of Table 1 - Unique antibody epitopes are associated with different conformational and AME effects for SARS-CoV-2.

mAb Group[a]	mAb Name	Conformational Regulation[b]	AME[c]	PDB ID[d]	Heavy/Light Chain Subclass[e]	Neutralizing	# shared residues/# total residues[f]
1	CV30	Up-exclusive[21]	None	*6XE1[21]	IGHV3-53, IGKV3-20[21]	Yes[21]	27/38
	C105	Up-exclusive[25]	None	6XCM[25]	IGHV3-53, IGLV2-8[25]	Yes[42]	27/34
	MW07	Up-exclusive[10]	None[g10]	*7DK2[10]	IGHV3-7, IGKV1-NL1[10]	Yes[10]	27/46
2	2-4	Down-exclusive[24]	None	6XEY[24]	IGHV1-2, IGLV2-8[24]	Yes[24]	19/20
	C144	Down-exclusive[19]	None	7K90[19]	IGHV3-53, IGLV2-14[19]	Yes[42]	19/27
3	DH1047	Up-exclusive[9]	FcγRI & FcγRIIb[h9]	7LD1[9]	IGHV1-46, IGKV4-1[9]	Yes[9]	Unknown/37
	DH1046	Unknown	FcγRI & FcγRIIb[h9]	n/a	IGHV3-23, IGKV1-5[9]	Yes[9]	Unknown
4	DH1041	Up-preferring[9]	FcγRIIb[h9]	7LAA[9]	IGHV3-7, IGLV1-40[9]	Yes[9]	17/28
	DH1043	Up-preferring[9]	FcγRIIb[h9]	7LJR[9]	IGHV1-69, IGKV3-20[9]	Yes[9]	17/33
	MW01	Unknown	FcγRIIb[g10]	*7DJZ[10]	IGHV1-69, IGKV3-15[10^]	Yes[10]	17/31
	MW05	Unknown	FcγRIIb[g10]	*7DK0[10]	IGHV1-69, IGKV3-15[10^]	Yes[10]	17/32

a. Antibodies grouped by conformational regulation (Groups 1 and 2) and AME (Groups 3-5)
b. Antibodies classified as up-exclusive if they were reported as only being able to bind the up RBD conformation, either through cryoEM or through structure alignment. Antibodies classified as down-exclusive if they were reported to only bind the down conformation in cryoEM studies. Antibodies classified as up-preferring if they were reported to prefer the up conformation in cryoEM studies but were not reported to require it in order to bind. Antibodies classified as unknown if no structure was determined or if the crystal structure of the antibody was not reported to exclude or prefer binding to up or down RBDs.
c. Receptor through which AME was reported for each antibody. Antibodies classified as unknown if it has, to our knowledge, never been tested for AME. Antibodies classified as none if it was tested and showed no evidence of AME. To our knowledge, CV30, C105, 2-4, and C144 were tested for AME for the first time in this work (see Figures 3 and A1).
d. PDB IDs for each antibody, if available. Structures were determined using cryoEM unless marked with an asterisk (*), which indicates the structure was determined using x-ray crystallography.
e. Heavy and light chain subclasses as previously reported, gathered using CoV-AbDab[43]. While the two antibodies marked with the up arrow (^) have the same variable (V) genes, as shown, they have unique heavy and light joining (J) genes[43].
f. Number of residues shared by all antibodies in the corresponding group/Number of residues in that antibody's full epitope
g. AME and FcγR-dependence confirmed in prior work using a combination of FcγR inhibitors, antibody mutations, testing on cell types with varying FcγR expression, and cell line engineering.
h. AME and FcγR-dependence confirmed in prior work using engineered cell lines expressing or lacking specific FcγRs.
